# The German version of the Quality of Marriage Index: Psychometric properties in a representative sample and population-based norms

**DOI:** 10.1371/journal.pone.0212758

**Published:** 2019-02-28

**Authors:** Tanja Zimmermann, Martina de Zwaan, Nina Heinrichs

**Affiliations:** 1 Department of Psychosomatic Medicine and Psychotherapy, Hannover Medical School, Hannover, Germany; 2 Department of Clinical Psychology, Psychotherapy, and Assessment, University of Braunschweig, Braunschweig, Germany; Chinese Academy of Medical Sciences and Peking Union Medical College, CHINA

## Abstract

The Quality of Marriage Index (QMI) is a 6-item internationally widely-used instrument assessing relationship satisfaction. This study aimed to evaluate (1) the psychometric properties of the German version in a representative sample of the German general population (*N* = 1431) as well as (2) effects of gender and age on relationship satisfaction. All participants were in a relationship. The German QMI demonstrates good item characteristics and excellent reliability (α = .94). The proposed one-factor solution was replicated. Differences in scoring on the QMI showed that males scored higher than females and differences between younger and older participants were found. The findings suggest that the German version of the QMI is suitable to reliably measure relationship satisfaction and may therefore be used as a brief screening instrument in a variety of settings and research questions. A cross validation in a sample of couples seeking help for relationship difficulties should be considered in future research. The limited number of items and the one-factor-solution do not suggest this instrument as a fine-tuned assessment tool for different dimensions of relationship satisfaction.

## Introduction

Relationship quality has been demonstrated to be an important indicator of adult, couple, and child well-being. Being in a happy marriage is related to better psychological and physical health. This effect depends mainly on the relationship quality and not on partnership status in isolation [1,2]. In contrast, divorce and relationship deterioration are omnipresent and provoke substantial mental and physical health costs on individuals and society [3]. Low relationship quality emerged as a significant risk factor for mental health symptoms [4]. For example, there is a high association between relationship distress and depression as well as substance abuse [5]. Moreover, relationship distress has been associated with higher incidence of both mental disorders [6] and poorer physical health [7]. The impact of relationship satisfaction is also reflected in a higher use of medical services of spouses in low quality relationships and diminished medical treatment adherence [8,9]. In addition, relationship quality is considered as an important impact factor on quality of life, e.g. for couples with chronic diseases such as cancer in the U.S. and Germany [10–12]. Thus, there is substantial need for (early) detection of deteriorating relationships before serious and irreversible damage has occurred.

Relationship quality is defined as a subjective and global evaluation of the relationship as well as behaviors in the relationship and can be measured in different ways [13]. The availability of psychometrically sound instruments to assess relationship satisfaction is central to clinical and basic relationship research. Moreover, reliable and valid instruments are essential in providing therapists with accurate information about the quality of the relationship of couples.

An often used measure to assess relationship satisfaction is the Dyadic Adjustment Scale [14] with 32 items. It is focused on relationship adjustment, and includes four subscales (dyadic satisfaction, dyadic cohesion, dyadic consensus, and affectional expression). It is also available in short versions: DAS-4 [15] or DAS-12 [16]. Research on the DAS in the U.S. and Germany points at the difficulty to replicate all four factors (e.g. [16]) while there is good evidence for a higher-order factor of adjustment [17]. As the DAS seems to be factorial invariant across gender, it was suggested that differences between men and women reflect indeed gender differences (instead of a measurement bias). However, the DAS was also criticized as having poor levels of precision when assessing relationship satisfaction in comparison to other measures [18].

Shorter scales such as the Quality of Marriage Index (QMI [19]), developed in the U.S., have been suggested as more convenient for measuring relationship satisfaction, compared to longer scales [20] and are recommended as a global assessment of relationship satisfaction [21]. The QMI’s brevity (6 items) may make the questionnaire preferential for therapists and clinical researchers who want to screen for relationship (dis-)satisfaction. The QMI asks participants to report the extent to which they agree or disagree with global statements regarding the quality of their relationship (e.g., “We have a good relationship”). The six items were selected from an original pool of more than 260 items [19]. Studies in which the scale has been utilized have consistently yielded very high internal consistency (i.e. alpha > .90) for couples (e.g. [3, 22, 23]). The German version of the QMI was developed by Zimmermann, Lause, and Heinrichs [24] on the basis of the English original version. Results from an exploratory factor analysis (EFA) in a convenience German sample of 848 married as well as cohabitant participants indicated a one-factor solution as proposed in the original English version. In addition good internal consistency was found [24]. However, an evaluation including item statistics, factorial structure using exploratory and confirmatory factor analysis (CFA), and internal consistency in a large population-based sample of the German general population is lacking. Furthermore, the development of norms is crucial for interpreting single scores, e.g. from couples seeking help for the individual or dyadic health. Therefore the first aim of the current study was to examine the psychometric properties of the German version of the QMI in a larger, population-based sample (in addition to a convenient sample).

In addition, measurement invariance across relevant subgroups was determined. In previous studies, males were shown to score significantly higher than females on the QMI indicating a higher relationship satisfaction [24] although it is unclear if this is due to a measurement bias or reflects a true difference between gender. Research on the influence of age on the QMI is also limited. Previous studies in German samples using the QMI showed no linear association between age and relationship satisfaction [24]. Similarly, one study using the DAS in Germany [16] did not find significant associations between the satisfaction subscale of the DAS and participant age. However another study in Germany using the Partnership Questionnaire (PFB) indicates a link between age and relationship satisfaction using age groups [25]. This may hint at non-linear associations of relationship satisfaction with age. Therefore, the second aim of the study was to evaluate the measurement invariance of the German version of the QMI across gender and age.

## Materials and methods

### Data sampling

Between January and March 2016, a representative sample of the German general population older than 14 years of age was recruited for a cross-sectional questionnaire survey (see ‘Field work’) with the assistance of a demographic consulting company (USUMA, Berlin, Germany). A random sampling procedure with three-stages was conducted: in the first step, a selection of 258 regional sampling areas was randomly selected (for more information see https://www.adm-ev.de/en/services/the-adm-sampling-system/); in the second step, a random procedure to select households of the respective area was implemented within all sampling areas; in the final step, one member of the selected household fulfilling the inclusion criteria was sampled randomly in a pre-specified standardized manner. Participants fulfill the inclusion criteria if they were older than 14 years, fluent in German, and provided written informed consent; for underage participants, parent or guardian consent was obtained. The sampling procedure is designed to yield randomly samples representative in terms of gender, education and age of the German population. The study was approved by the ethics committee of the University of Leipzig, Germany (Az 452-15-21122015).

### Field work and measures

Selected individuals were approached in-person by a trained interviewer. Participants were informed about the study and provided written informed consent. Interviewers collected sociodemographic information face-to-face. Afterwards, participants filled out a battery of self-report questionnaires, including the German version of the Quality of Marriage Index [19] with six items. Respondents answer the first five items on a 7-point scale ranging from 1 (*strongly disagree*) to 7 (*strongly agree*). Examples of these items include, “we have a good relationship”, and “my relationship with my partner makes me happy”. The sixth item asks participants to rate their overall level of happiness on a 10-point scale ranging from 1 (*extremely low*) to 10 (*extremely high*). The sum of the items was used, with a possible range from 6 to 45. Higher scores indicate a higher relationship satisfaction. According to a prior German survey, a cutoff score of 34 or higher defines individuals as being satisfied with their relationship [24].

### Participants

A total of 4902 households were randomly sampled. Of these, 2524 individuals participated in the large survey (51.5% response rate). Only individuals in a relationship were asked to fill out the QMI, therefore a final sample of *N* = 1431 participants emerged. Table 1 displays sociodemographic characteristics of the sample. All participants were in a relationship. For subsequent cross-validation analyses the sample was divided randomly into two subsamples by applying stratified probability sampling with consideration of gender and age using SPSS 24 random case selection procedure. During this procedure the representative characteristics of the population based survey approximately remained within each subsample. The randomized division of this large sample into two subsamples was necessary to be able to first create a model and then test the model fit with an independent, separate data set. No significant differences were found for the two subsamples (Table 1).

**Table 1:** Sociodemographic characteristics of the total sample and the two subsamples.

	Total sample (*N* = 1431)	Subsample I (*N*_1_ = 708)	Subsample II (*N*_2_ = 723)
Age in years (SD, range)	49.3 (15.5, 16–91)	48.6 (15.9, 16–86)	50.0 (15.2, 16–91)
Age groups in years (%)			
14–30	186 (13.0)	103 (14.6)	83 (11.5)
31–60	885 (61.8)	430 (60.7)	455 (62.9)
> 60	360 (25.2)	175 (24.7)	185 (25.6)
Gender (%)			
male	687 (48.0)	333 (47.0)	354 (49.0)
female	744 (52.0)	375 (53.0)	369 (51.0)
Relationship status (%)^1^			
married/cohabiting	1045 (73.3)	508 (71.7)	537 (74.8)
non-married	245 (17.2)	136 (19.2)	109 (15.2)
divorced	109 (7.6)	53 (7.5)	56 (7.8)
widowed	27 (1.9)	11 (1.6)	16 (2.2)
Nationality (%)			
German	1366 (95.5)	671 (94.8)	695 (96.1)
other	65 (4.5)	37 (5.2)	28 (3.9)
Education (%)			
< 12 years	1114 (77.8)	540 (76.3)	574 (79.4)
≥ 12 years	317 (22.2)	168 (23.7)	149 (20.6)
Employment status (%)			
Not unemployed	1356 (95.6)	675 (96.2)	681 (95.0)
unemployed	63 (4.4)	27 (3.8)	36 (5.0)
Monthly household income (%)			
< 1250 EUR	88 (6.3)	42 (6.1)	46 (6.6)
1250—< 2500 EUR	548 (39.5)	285 (41.5)	263 (37.6)
≥ 2500 EUR	750 (54.1)	360 (52.4)	390 (55.8)

*Notes*. Percentages are calculated from valid cases. No significant differences were found for the two subsamples.

^1^ all participants were in a relationship.

### Statistical analysis

All statistical analyses were conducted using SPSS 24. All tests were based on a significance level of 0.05. For evaluating the internal factor structure of the QMI as an indicator of construct validity means of a split-half factor analysis approach were conducted. SPSS 24 random case selection procedure was used to split the total sample randomly into two subsamples. In the first step, the data were analyzed in the first split-half sample by a Principal Axis Factor analysis (PAF), with varimax rotation [26]. Extraction criteria were eigenvalues > 1 in conjunction with a visual inspection of the scree plot. In the second split-half sample a CFA was performed examining the model obtained in the EFA using AMOS 24. As absolute fit indices, the Standardized Root Mean Square Residual (SRMR) and the Root Mean Square Error of Approximation (RMSEA) including the 90% confidence interval were used. As comparative fit indices the Comparative Fit Index (CFI) and the Tucker-Lewis Index (TLI) were calculated. SRMR values < .08 indicate a good model fit; RMSEA values below.08 with a significance value below.05 indicates acceptable fit. CFI and TLI ≥ .90 indicate a good model fit, values above.95 an excellent fit [27,28].

Item descriptives, item difficulties as well as corrected item-total-correlation were examined. Internal consistency reliability coefficients were evaluated for the sample.

Effects of gender and age on QMI scores were assessed using analysis of variance (ANOVA). Partial η^2^ was calculated as estimation of effect sizes with values of.01 considered as small,.06 as medium and.14 as large effects.

A receiver operating characteristic (ROC) curve was applied to calculate the cutoff gap for QMI and the area under the curve (AUC) to represent accuracy. For this purpose, item 6 of the QMI was dichotomized (1 = *unhappy to rather happy*, 2 = *happy to perfectly happy*). The AUC provides information about the discrimination ability of the test with scores >.90 for excellent test, >.80 good, and >.70 fair [29]. In addition, sensitivity (SEN), specificity (SPE) and the Youden-Index (J = SENE + SPE-1) were calculated.

## Results

### Internal factor structure

For the first analysis a subsample of *N*_1_ = 708 participants of the total sample were used; 375 (53%) were female. The average age was 48.6 years (*SD* = 15.9, range = 16–86; Table 1). The various indicators of factorability were good (Kaiser-Meyer-Olkin index = .93; Bartlett’s test of sphericity: Chi^2^ = 4388.1, df = 15, *p* < .001), and the residuals indicate a good solution. One factor with an eigenvalue of greater than 1.0 was found; the scree plot also indicated one factor. The one factor (eigenvalue = 4.88) explained 81.3% of the variance and replicated the original one factor solution of the QMI in a German sample. All factor loadings were ≥ .72.

**Fig 1:**
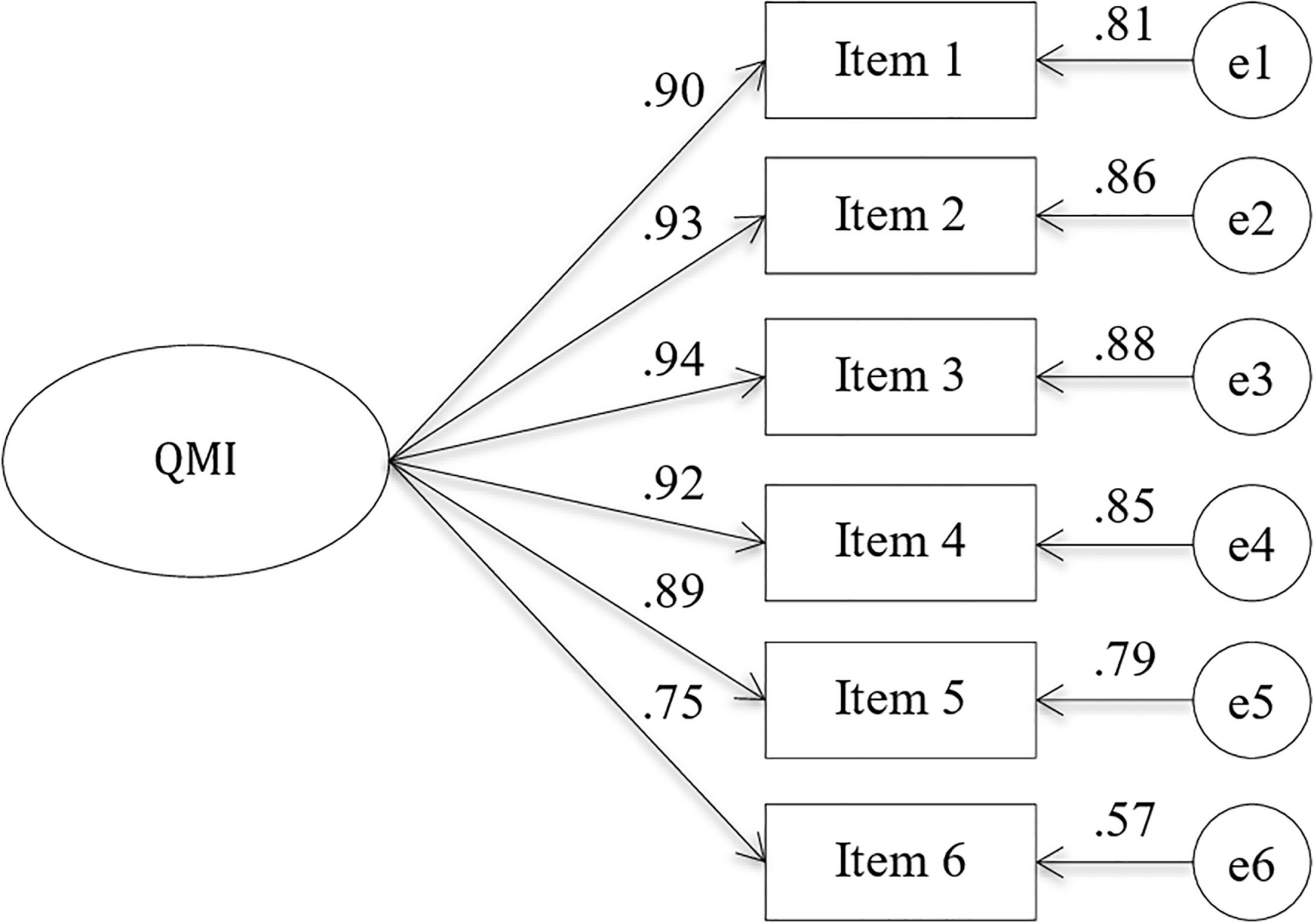
Results of the confirmatory factor analysis, study II (n_2_ = 723). RMSEA = .11, CFI = .99, TLI = .98, and SRMR = .01.

For the CFA the second randomly selected subsample (*N*_2_ = 723) was included. These participants were not included in the sample of the EFA analysis. The average age was 50.0 years (*SD* = 15.2, range 16 to 91), and *n* = 369 (51%) were female (Table 1). The CFA confirmed the one-factor solution for the QMI (RMSEA = .11, CFI = .99, TLI = .98, and SRMR = .01). The RMSEA was out of the acceptable range and the chi^2^ was significant, chi^2^(9) = 77.02, *p* < .001. The completely standardized loadings and the standardized residuals are shown in Fig 1.

### Item analysis and reliability

The item characteristics of the German QMI are displayed in Table 2. All items were negatively skewed and showed a positive kurtosis. Item difficulties ranged between 80% and 88% indicating a high probability of scores > 6 (“strongly agree”). Corrected item-total-correlations were high (.72–.90). Cronbach’s alpha was.94 indicating a high reliability.

**Table 2:** Factor loadings and item characteristics of the QMI (N = 1431).

No.	Original Item (German version)	PAF Factor I (*N*_1_ = 708)	Mean	SD	*p*_i_ (%)	*r*_it_
1	We have a good relationship. (Wir haben eine gute Partnerschaft.)	.92	6.2	.95	86.7	.87
2	My relationship with my partner is very stable. (Meine Beziehung mit meinem Partner ist sehr stabil.)	.92	6.2	1.1	87.3	.87
3	My relationship with my partner is strong. (Unsere Partnerschaft ist stark.)	.94	6.2	1.1	86.5	.90
4	My relationship with my partner makes me happy. (Meine Beziehung mit meinem Partner macht mich glücklich.)	.92	6.2	1.1	86.0	.88
5	I really feel like part of a team with my partner. (Ich fühle mich wirklich wie ein Teil eines Teams mit meinem Partner.)	.91	6.1	1.2	85.2	.86
6	All things considered, what degree of happiness best describes your relationship? (Wie glücklich schätzen Sie auf einer Skala von 1 = sehr unglücklich bis 10 = perfekt glücklich Ihre Partnerschaft–über alles betrachtet—ein?)	.78	8.2	1.8	80.2	.72

*Notes*. QMI = Quality of Marriage Index; Mean of each item (range 1–7), PAF = principal axis factor analysis; SD = Standard deviation; *p*_i_ = item difficulty; *r*_it_ = corrected item-total correlation. Respondents answer the first five items on a 7-point scale ranging from 1 (*strongly disagree*) to 7 (*strongly agree*). The sixth item participants rate their overall level of happiness on a 10-point scale ranging from 1 (*extremely low*) to 10 (*extremely high*).

*N*_1_ = subsample I drawn by random case selection procedure using SPSS 24

### Effects of gender and age on relationship satisfaction

In addition, we analyzed the effect of age and gender for the QMI in this large, representative sample (*N* = 1431). The linear correlation between age and QMI was not significant (*r* = .04, *p* = .06). However, a two-way-between-subjects ANOVA using gender and age groups (14–30, 31–60, >60 years) showed significant effects for age groups (*F*(2, 1425) = 5.98, *p* < .01) as well as for gender with males showing slightly higher relationship satisfaction (*M* = 39.49, *SD* = 5.81) than females (*M* = 38.65, *SD* = 6.91; *F*(2, 1425) = 4.74, *p* < .05). The post-hoc analysis indicates that participants aged 31 to 60 (*n* = 885; *M* = 38.58, *SD* = 6.70) scored significantly lower on the QMI than participants age 61 and older (*n* = 360, *M* = 40.09, *SD* = 5.18). A closer inspection of this age effect demonstrated that participants aged 35 to 44 (*n* = 256, *M* = 38.05, *SD* = 7.57) showed significant lower relationship satisfaction than participants in the age of 65 to 74 (*n* = 181, *M* = 40.35, *SD* = 4.69, *F*(6, 1424) = 3.16, *p* < .01).

**Fig 2:**
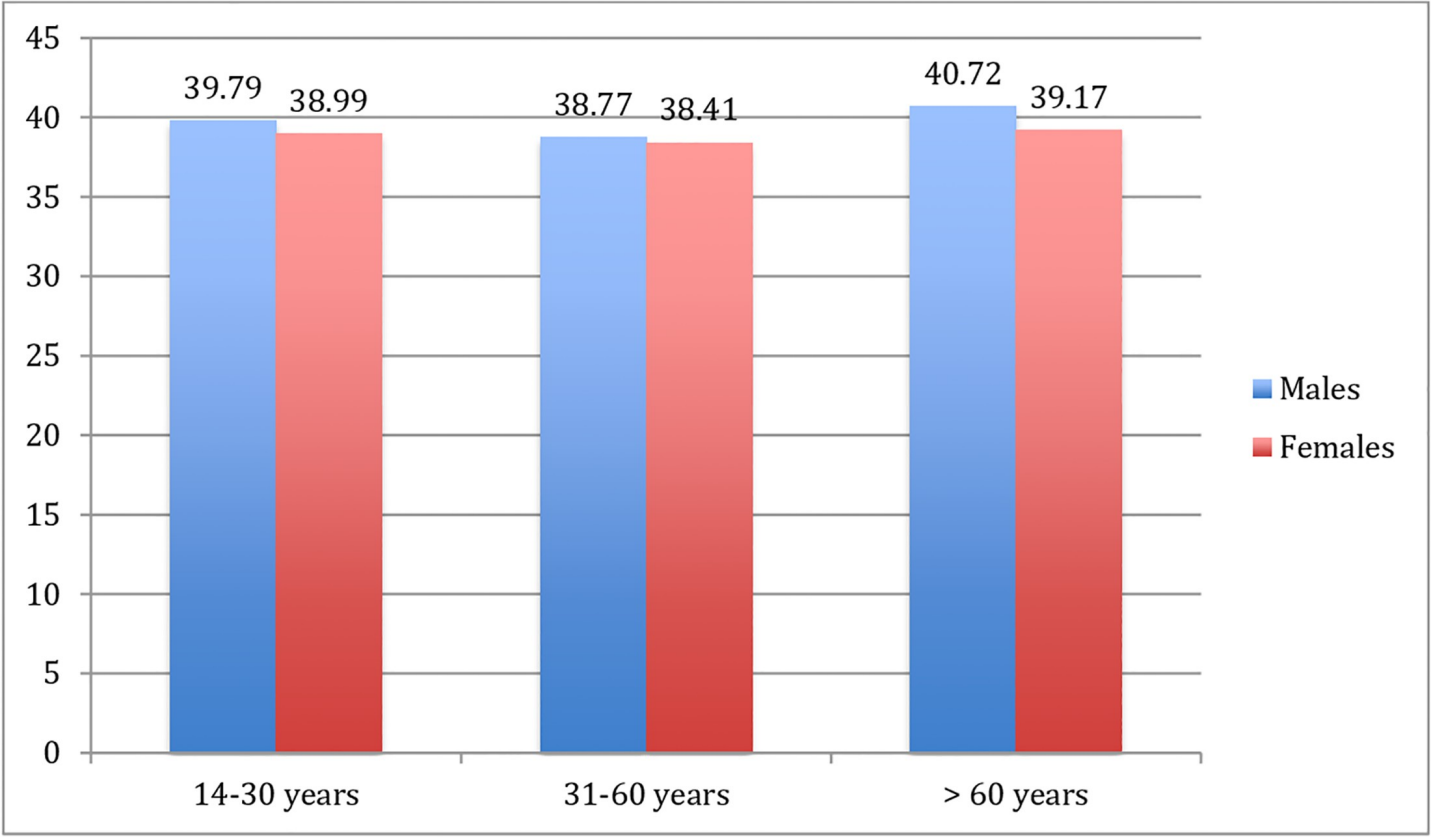
QMI total scores for age and gender groups. (males: n = 75 (14–30 years), n = 401 (31–60 years), n = 211 (> 60 years); females: n = 111 (14–30 years), n = 484 (31–60 years), n = 149 (> 60 years)).

All effect sizes are small for both, gender and age. There was no significant interaction between these age groups and gender. Fig 2 demonstrates the relationship between age and gender for the QMI total score.

### Cutoff scores

Cutoff scores were calculated in the total sample as well as for males and females separately. The ROC analysis of the total sample showed an AUC of excellent fit (AUC = .96; 95% CI = .95–.98) resulting in a cutoff score of 34, with a sensitivity of 88%, specificity of 85%, and Youden-Index *J* = .73. For males, as well as for females, a cutoff score of 34 emerged (males: SEN = .92, SPE = .83, AUC = .96, 95% CI = .94–.98; females SEN = .89, SPE = .85, AUC = .96, 95% CI = .94–.98).

## Discussion

The purpose of the present study was to examine the psychometric properties of the German version of the QMI in a representative sample as well as effects of gender and age on relationship satisfaction. The QMI which was developed by Norton [19], is an internationally extensively used instrument assessing relationship quality. The six-item German version showed high internal consistency. The construct validity of the German version of the QMI was examined using PAF and CFA. Results from the EFA indicated a one-factor solution which replicated the original factor structure and explained 81.3% of the variance. The one-factor model showed a good to excellent fit in a CFA confirming the one-factor structure of the original version and supporting preliminary results from a first examination of the German version [24]. These results suggest that the QMI is a very homogenous measure that may be used if one is interested in a global, uni-dimensional evaluation of relationship satisfaction.

Men estimated their relationship quality significantly higher than women. This is in line with previous research on the German QMI in a non-representative sample [24] as well as studies using other short instruments to measure relationship quality [25]. While these findings are also consistent, more broadly, with research that has established that females report lower relationship satisfaction and quality [30–32], the effects are in general small, and it is also unclear if they reflect true gender differences or are produced by a measurement bias, at least with the present instrument. From the DAS there is some evidence suggesting these gender differences reflect true differences in the evaluation of relationship quality [17].

Similarly, we found small but significant effects of age groups on relationship satisfaction. These are, however, not linear but seem rather curvilinear. Specific life stages are linked to different challenges (e.g., raising a child in younger to middle age)., Role shifts due to the limited future time and decreasing contact with former colleagues and friends may lead to a higher significance of relationship quality for well-being in older age [1]. This is in line with research showing a curvilinear pattern over the life span, declining in the earlier years of marriage and increasing through the later years [33] and contrary to other studies indicating a decline of relationship satisfaction over time [34].

The cutoff scores determined through ROC analysis on the basis of the “Terman-Item” (Item 6 of the QMI) showed good sensitivity and specificity scores but should be considered with caution because the external criterion was a single item and this single item is also part of the total score. Further analyses of the discriminant validity are therefore needed using a truly external and also clinical relevant criterion (e.g., the Oral History Interview [35]). The revealed cutoff score of 34 is higher than the recommended cutoff of 29 of the original U.S. questionnaire [14,36] indicating that Germans scored higher on the QMI than the original population in the U.S. and that the score which divides those who are satisfied to those who are unsatisfied with their relationship is similarly higher in the German sample.

A major strength of the current study is the use of a large, population-based sample representative of the German general population with regard to gender and age. To our knowledge this is the first study examining the factor structure of the German version of the QMI in large sample using a confirmatory approach and to analyze effects of gender and age. Major limitations of the study concern the fact that no data on retest-reliability, criterion validity lack of information on external criteria, and the comparison of the QMI with existing measures of relationship quality were collected. Therefore, important other indicators of psychometric quality were not evaluated. The cross-sectional design of the study does not allow for conclusions about the direction of obtained association and precludes the assessment of measurement invariance over time. Moreover, the study did not include a clinical sample (e.g., couples in couple therapy or participants with mental disorders) although the (high) prevalence of these experiences in the general population (e.g. for depressive symptoms) suggest that a significant proportion of those individuals are also included in the present sample. These shortcomings should be addressed in further investigations.

In conclusion, the current study demonstrated that the 6-item German version of the QMI has adequate psychometric properties and reliably measures relationship quality across gender and age. The provided cutoff scores may be used for assessment purposes, particularly in group assessments with this screening instrument. For individual, in-depth assessment of relationship quality, including various dimensions, and a more comprehensive clinical assessment, the QMI may not be the preferred choice.

## Supporting information

S1 Dataset (XLSX)
